# 1383. Malaria Prophylaxis Use In a Travel Clinic, How Are We Doing?

**DOI:** 10.1093/ofid/ofad500.1220

**Published:** 2023-11-27

**Authors:** Dennis Hogan, Eliahu Bishburg, Madhu Suryadevara

**Affiliations:** RWJBH NEWARK BETH ISRAEL MEDICAL CENTER, Princeton, New Jersey; Newark Beth Israel Medical Center, Newark, New Jersey; RWJBH - Newark Beth Israel Medical Center, Newark, New Jersey

## Abstract

**Background:**

Malaria chemoprophylaxis is indicated for travelers going to geographic areas designated as endemic for malaria. Often persons traveling to their native countries where malaria is present consider themselves immune and decline chemoprophylaxis. This study aimed to evaluate malaria chemoprophylaxis utilization in a travel clinic and whether it is correlated with the patient’s native place.

**Methods:**

A retrospective study of patients attending a travel clinic between 2021 to 2023. Data were extracted on demographics, trip destinations, trip duration, and, malaria chemoprophylaxis prescribed. Pharmacies were contacted to verify prescription filling. Patients admitted with malaria were analyzed at the same time period. Comparison between groups was made using a chi-square test. p < 0.05 is considered significant.

**Results:**

One hundred patients were included. Median age 55.5 years (21-80). 62 % (62) were females while 38% were males. There were ninety-four 94 travelers to Africa, 6 to South America, and 1 to Asia. Of the travelers, 44 were US natives while 56 were non-US natives. The median trip duration was 14 and 15 days among US and non-US natives respectively. All 100 patients received prescriptions for malaria prophylaxis: Atovaquone/Proguanil was prescribed to 94, while 6 were given mefloquine. Of the 100 prescriptions, 58 were filled. Of the 44 travelers who were US natives,33/44 (75 %) filled their prescriptions. Among the 56 non-US natives, 25/56 (44 %) filled prescriptions, p -0.002. During the study period, 10 patients (6 females and 4 males) were admitted with a diagnosis of malaria in returning travelers, median age of 54.5 years. The median trip duration was 19 days. All were non-US natives returning from Africa (Nigeria, Ghana, Guinea, and Mali). Only 1/10 patients took mefloquine chemoprophylaxis. All the patients were treated successfully without sequelae.

**Conclusion:**

In our travel clinic malaria chemoprophylaxis was prescribed to all patients as indicated, US native patients filled their prescriptions significantly more than nonnative patients. Malaria in returning travelers was diagnosed exclusively in non-US natives who did not take malaria chemoprophylaxis.

**Disclosures:**

**All Authors**: No reported disclosures

